# Influence of Bioactive, Resin and Glass Ionomer luting cements on the fracture loads of dentin bonded ceramic crowns

**DOI:** 10.12669/pjms.36.3.1946

**Published:** 2020

**Authors:** Fahim Vohra, Manea Altwaim, Abdulaziz S Alshuwaier, Modhi Al Deeb, Yasser Alfawaz, Mohammed Alrabiah, Tariq Abduljabbar

**Affiliations:** 1 Fahim Vohra, Department of Prosthetic Dental Science, College of Dentistry, King Saud University, Riyadh 11545, Saudi Arabia; 2 Manea Altwaim, Intern, Department of General Dentistry, College of Dentistry, King Saud University, Riyadh 11545, Saudi Arabia; 3 Abdulaziz S Alshuwaier, Intern, Department of General Dentistry, College of Dentistry, King Saud University, Riyadh 11545, Saudi Arabia; 4 Modhi Al Deeb Department of Prosthetic Dental Sciences, College of Dentistry, King Saud University, Riyadh 11545, Saudi Arabia; 5 Yasser Alfawaz, Department of Restorative Dental Sciences, College of Dentistry, King Saud University, Riyadh 11545, Saudi Arabia; 6 Mohammed Alrabiah, Department of Prosthetic Dental Sciences, College of Dentistry, King Saud University, Riyadh 11545, Saudi Arabia; 7 Tariq Abduljabbar, Department of Prosthetic Dental Sciences, College of Dentistry, King Saud University, Riyadh 11545, Saudi Arabia

**Keywords:** Bioactive cement, Dentin bonded crowns, Failure load, Glass ionomer cement, Resin cement

## Abstract

**Objective:**

To investigate the failure loads of dentin bonded all-ceramic crowns when luted with Bioactive, resin and glass ionomer cements (GIC) in an in-vitro setting.

**Methods:**

This study was conducted at King Saud University, Saudi Arabia, from Nov.2018 to March 2019. In this study, 60 premolar teeth were prepared for dentin-bonded ceramic crowns. Lithium disilicate ceramic crowns fabricated using CAD-CAM technique were cemented to teeth using Bioactive (ACITVA), Resin (Nexus 3 Gen) and GIC (Ketac Cem- Maxicap). Half of the bonded specimens in each group were thermocycled (50000 cycles), however the remaining half were not aged (n=10). Fracture loads of bonded crowns were assessed by exposing them to static axial occlusal loads (1mm/min) using a round ended metal probe in a Universal testing machine. Means and standard deviations among the study groups were compared with ANOVA and Tukey-Kramer multiple comparisons test.

**Results:**

Highest failure loads were observed in resin group without ageing (thermocycling) (689.13±89.41 N), however, the lowest loads were observed in GIC specimens with ageing (243.16±49.03 N). Among non-aged samples, failure loads for Bioactive (480.30±47.26 N) group were less than Resin (689.13±89.41 N) samples but higher than GIC (307.51±45.29 N) specimens respectively. Among the aged specimens, Bioactive (404.42±60.43 N) showed significantly higher failure loads than GIC (243.16±49.03 N), however lower failure loads than Resin (582.33±95.95 N) samples.

**Conclusions:**

Dentin boned crowns with resin cementation showed higher failure loads than Bioactive and GIC luted crowns. Crowns luted with Bioactive cement showed acceptable failure loads for use as restoration on anterior teeth.

## INTRODUCTION

Oral rehabilitations require cementation of provisional and definitive restoration in the form of crowns, veneers, fixed partial dentures and frameworks on teeth and implants.^1^ Cementation of indirect restorations is critical for the long-term prognosis of restorations as it seals dead space (cement space), provides retention; prevents microleakage and secondary caries.^2^ For cementation of contemporary highly translucent esthetic ceramic crowns and veneers, resin cements are employed for improved adhesive bond, esthetic outcome and support of delicate ceramic shells.^3^ However, resin cements fail to create a chemical bond with the dentinal surface (hydrophobic) and show minimum release or absorption of fluoride and calcium ions, hence are associated with post cement sensitivity and microbiological activity.^4^ Dentin-bonded all-ceramic crowns (DBC) are full coverage restorations bonded to underlying dentin and enamel using resin luting materials.

Continued developments in restorative materials have introduced bioactive luting agents for restorative cementation. These materials (ACTIVA) are based on a resin matrix with hydrophilic ions, which allow for uptake and release of calcium, fluoride and phosphate ions in reaction to the changes of pH in the oral environment.^5^ This bioactivity allows Bioactive cements to enhance the chemical bonds between cement and dentin, reduce microleakage, improve durability and tooth remineralization.^6^-^8^ It is proposed, that the release of bioactive molecules like calcium silicate stimulates aperture sealing and apatite formation potentially stabilizing restorative interface and improving clinical success.^9^

The reduced microleakage of Bioactive cements is reported to be comparable to resin cements.^10^ As bonded ceramic crowns and veneers enhance their fracture resistance through adhesive bonding to tooth, ability of bioactive cements to provide mechanical support to dentin bonded crowns and veneers is critical for clinical restorative success. In a study by Girn et al., compressive and tensile strengths of bioactive materials were shown to be comparable to resin materials.^11^ In similar studies, flexural strength and flexural fatigue of these materials were lower than conventional resins.^11^,^12^ Therefore, whether bioactive cements enhance fracture resistance of bonded ceramic restorations is controversial. It is hypothesized that Bioactive cements when employed as a luting agent for dentin bonded ceramic crowns will show comparable failure/fracture loads to resin luted crowns under standardized protocol. The aim of the study was to investigate the failure loads of dentin bonded all-ceramic crowns when luted with Bioactive, resin and glass ionomer cements (GIC) in an in-vitro setting.

## METHODS

This study was conducted at King Saud University, Saudi Arabia, from November 2018 to March 2019 after the approval of ethical committee (Ref. No. IR 0331). In this study, 60 human maxillary premolars were collected after orthodontic extractions. The teeth were cleansed and stored in 0.1% thymol solution (Thymol, Fisher Scientific, NJ, USA). All teeth were mounted in orthodontic acrylic resin (Orthodontic Resin, Dentsply caulk, DE, USA) vertically, 2mm below the cemento-enamel junction (CEJ) using a polyvinyl carbonate section.

### Tooth Preparations

Each tooth was prepared to standard dimensions, for a complete coverage all ceramic dentin bonded crown. The amount of tooth reductions included, occlusal preparation of 1.7mm, a rounded shoulder of 1mm, axial preparation of 1.5mm and 8-degree taper. A single experienced operator (FV) prepared the teeth using a putty index (Polyvinyl siloxane- Aquasil Putty-Dentsply-sirona-MN-USA) of individual teeth. The tooth height was nearly 4.5mm with rounded line angles and margins 0.5mm below the CEJ. Using customized resin trays, impressions of the prepared teeth were recorded using light body VPS (vinyl polysiloxanes) (Imprint, 3M ESPE, MN, USA) on the tooth preparation and regular body VPS (Imprint, 3M ESPE, MN, USA) in the trays. Impressions were poured with die stone (SheraPure Diestone, Auckland, New Zealand) and dies were prepared for scanning.

### Specimen Fabrication

Dentin bonded crowns were fabricated using lithium disilicate ceramics (IPS-Emax-CAD- Ivoclar Vivadent, NY, USA). Each stone die was sprayed with contrast spray (IPS Labside, Contrast spray, Ivoclar Vivadent, NY, USA). Each die was scanned using CAD-CAM scanner (Ceramill Map 400, Amann Girrbach, NC, USA), on the STL (Standard Triangle Language) file crowns with occlusal cuspal contours (2mm cusp height, 1.5mm occlusal fissure thickness) and 1mm thickness axial surfaces were designed with EvoCad (Design software, Amann Girrbach, NC, USA). Emax CAD (lithium disilicate) was milled (Ceramill motion 2, Amann Girrbach, NC, USA) to the required dimensions and a single experienced technician performed all laboratory procedures. A cement space of 0.02 mm at 2mm from the prepared margin was incorporated. All specimens were etched with 9.5% hydrofluoric acid (HF Acid- Ceram-Etch Gel Gresco products, TX, USA) for 30 seconds (sec). And a single application of silane (Monobond S, Ivoclar Vivadent, NY, USA) was applied with a microbrush and allowed to dry.

### Cementation

Fabricated crowns were assessed for thickness and were stored at room temperature (20 °C). All 60 dentin bonded ceramic crowns were randomly assigned to three cement groups. Group-1 (n=20): Bioactive (Activa Bioactive cement- ACTIVA Pulpdent, MA, USA); Group-2 (n=20): Resin (positive control) (Nexus 3- Third Generation, Kerr, CA, USA) and Group-3 (n=20): Glass Ionomer Cement (negative control) (GIC- Ketac Cem- Maxicap- 3M ESPE, MN, USA). Each crown was cemented to the corresponding tooth preparation with the assigned cement following manufacturer’s instructions. Equal amount of cement was dispensed in each crown and smeared with the walls using a plastic instrument. Crowns were cemented on tooth preparation at a standard load of 10 N for 1 minute. Excess cement was removed using a microbrush and discoid-cleoid carver. For the specimens in the Group-2 (resin group), tooth surface was etched (phosphoric acid- Caulk 34% Tooth Conditioner Gel, DENTSPLY Caulk, DE, USA) for 15 sec, washed and dried. A bonding agent with primer and adhesive (Prime & bond NT, DENTSPLY Caulk, DE, USA) was applied to the tooth with a micro brush for 20 secs, dried with air (5 sec) and photopolymerized (Bluephase ® C8, Ivoclar Vivadent, Schaan, Liechenstein-650 mWcm-^2^) each surface for 20 secs (occlusal, buccal, lingual, mesial and distal). For specimens in Group-1 (Bioactive), tooth preparation was dried with air for 5 sec and crowns cemented followed by removal excess and photo-polymerization similar to Group-2 specimens. In the specimens cemented with GIC, cementation followed the standard protocol however, a layer of petroleum jelly was applied to the margin to prevent water dissolution or absorption during cement setting. All specimens were stored in distilled water at 37°C for 24 hours.

Half of the samples in each cement group (n=10) were thermocycled (TC) (50000 cycles) (SD Mechatronik, Thermocycler, GMBH, Miebacher Strabe, Germany) between 5 and 55ºC water baths (dwell time 30 sec). The remaining half (n=10) specimens in each cement group were not aged. This resulted in a total of 6 study groups, ten in each cement group, with and without TC.

### Failure testing

**Fig.1:**
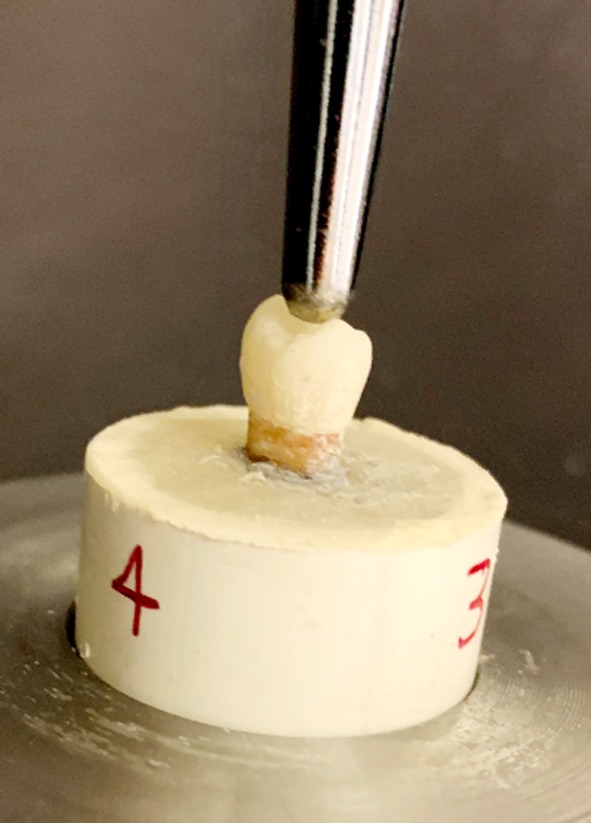
Load application assembly for crown failure test.

All study specimens were subjected to occlusal load using hydraulic universal testing machine (Model 4411; Instron Corp, Canton, Mass). A static load was applied to with a round head stainless steel probe contacting both lingual and buccal cusp slopes. Load was applied at a crosshead speed of 1 mm/min until failure (Fig.1).

Acquired failure loads among the study groups were assessed for normality using Kolmogorov-Smirnov test. Means and standard deviations among the groups were compared suing ANOVA and Tukey multiple comparisons test. A p-value of less than 0.05 was considered significant.

## RESULTS

The data obtained for failure loads in newton (N) was normally distributed. Highest failure loads were shown by samples in resin group without ageing (thermocycling) (689.13± 89.41 N), however, the lowest loads on fracture were displayed by crowns cemented with GIC with ageing (243.16 ± 49.03 N).

**Fig.2:**
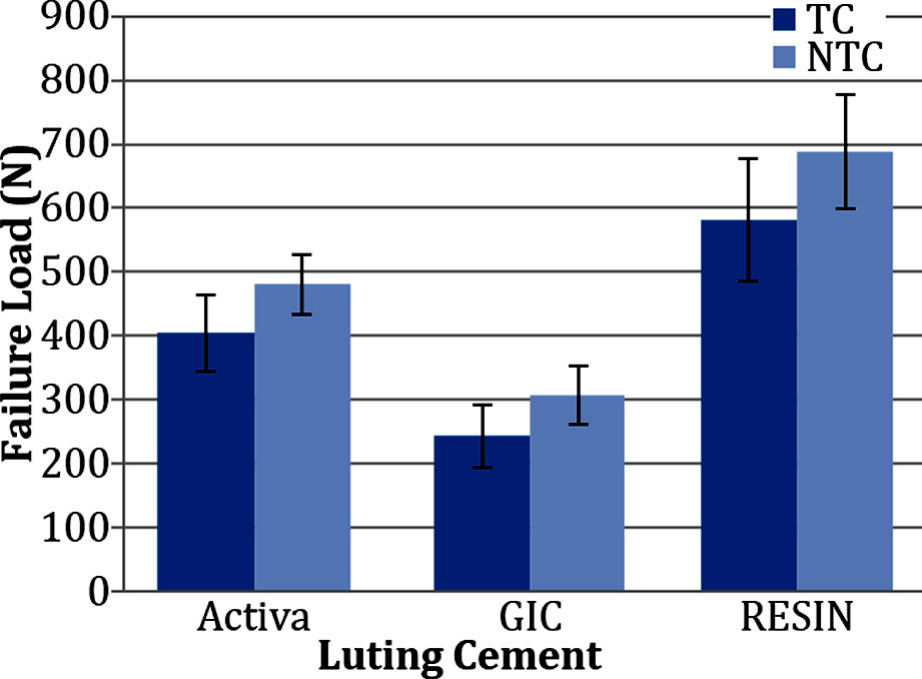
Graphical comparison of mean and SD for fracture loads in specimens among tested groups. Activa; Bioactive Cement, GIC; Glass ionomer cement, Resin; Nexus 3^rd^ Gen Luting cement.

Specimens not exposed to ageing showed significant difference in their failure loads, with Resin specimens (689.13± 89.41 N), showing significantly higher values (p<0.01) compared to both Bioactive (480.30± 47.26 N) and GIC (307.51± 45.29 N), samples (Fig.2). Failure loads for Bioactive group were less than Resin samples but higher than GIC specimens respectively. Samples among the GIC groups presented significantly lower failure loads compared to both Resin and Bioactive groups (p<0.01) (Table-I).

**Table-I:** Failure loads of ceramic crowns cemented with Activa, GIC and Resin cements with and without thermocycling.

Study Groups	Thermocycled		P- value*	Non-Thermocycled		P-value*
	
	Mean^$^	SD		Mean^$^	SD	
Activa	404.42^Aa^	60.43	0.001	480.30^Ab^	47.26	0.001
GIC	243.16^Ba^	49.03		307.51^Bb^	45.29	
Resin	582.33^Ca^	95.95		689.13^Ca^	89.41	

SD: Std. Deviation.

* ANOVA.

$ Tukey multiple comparisons test.

Dissimilar superscript capital alphabet in same column denotes statistical difference.

Dissimilar superscript small alphabets in same row denote significant difference.

Specimens exposed to ageing showed a similar pattern for failure load values as un-aged samples among their respective cement groups (Fig.2). Crowns cemented with Resin cement (582.33 ± 95.95 N) showed significantly higher (p< 0.01) failure loads than Bioactive (404.42 ± 60.43 N) and GIC (243.16 ± 49.03 N) samples. Bioactive crowns showed significantly higher failure loads than GIC, however lower failure loads than Resin samples. GIC samples showed significantly lower failure loads than both Resin and Bioactive samples respectively (p< 0.01) (Table-I).

**Fig.3:**
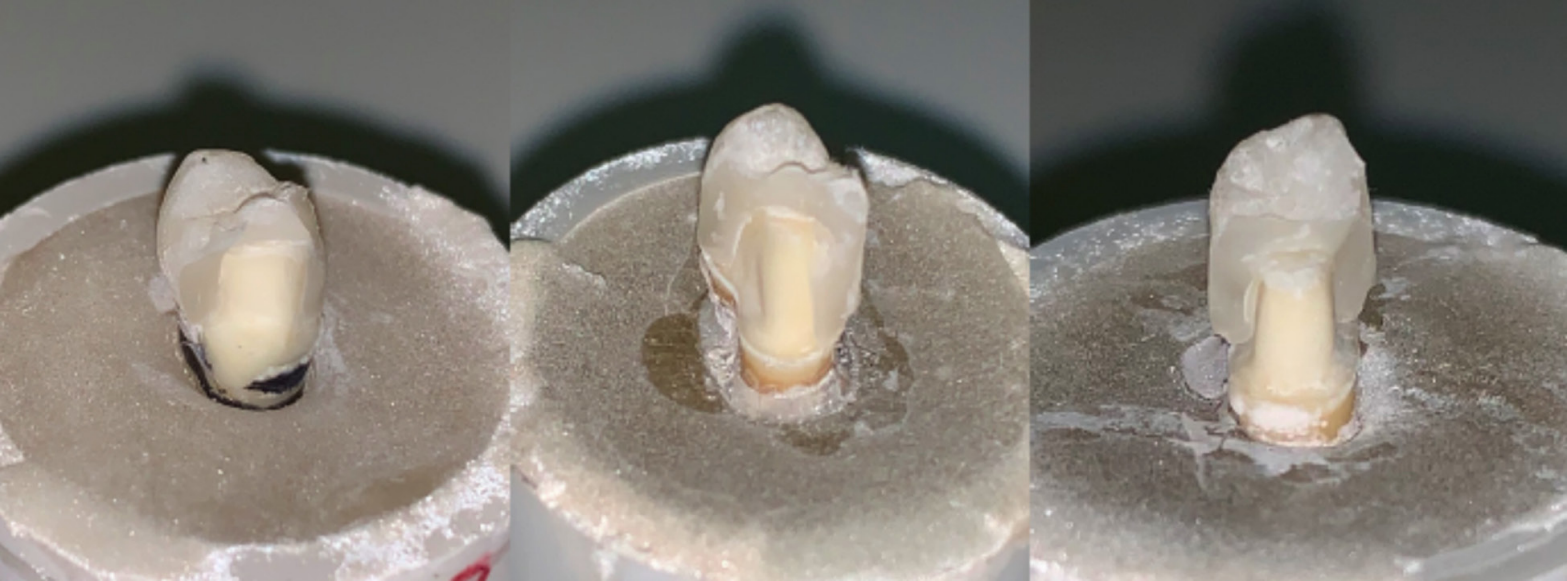
Failure modes of specimen crowns among study groups. A. Resin; B. ACTIVA; C. GIC.

Ageing through thermocycling showed a significant negative influence in reducing the failure loads values of GIC and Bioactive specimens (p<0.01). However, failure loads for Resin cemented crowns with and without ageing showed statistically comparable outcomes (p>0.01). Failure modes among the crowns, showed 100% of partial failures (cohesive failure in ceramic) within ceramic in the Bioactive and Resin specimens. However, among the GIC specimens, 65% (13 specimens) showed complete fracture of ceramic crowns (failure at the adhesive interface of ceramic and cement, with complete fracture of ceramic crown) exposing tooth structure (Fig.3 A, B, C).

## DISCUSSION

The present study was based on the hypothesis that, Bioactive cements when employed as a luting cement for dentin bonded ceramic crowns will show comparable fracture loads to resin luted dentin bonded crowns. The experiments revealed that failure loads for specimens cemented with Bioactive cement were significantly lower than those luted with resin cement (positive control). In addition, crowns cemented with GIC (negative control) showed significantly lower failure loads compared to both Bioactive and resin cement samples.

A myriad of influencers effect the failure loads of luted ceramic crowns, including, ceramic material, ceramic thickness, fabrication technique bonding substrate, bonding agent, storage conditions and load application.^13^,^14^ Experimental conditions were standardized as all crowns were fabricated with lithium disilicate ceramic having similar dimensions (1mm axial-dentin bonded crowns)^13^ fabricated using CAD-CAM technique.^15^ In addition, the cement space, bonding regime and protocols were according to manufacturer’s instructions to simulate clinical techniques.^16^ Tooth preparation (FV) and laboratory protocols and procedures (MT) were performed by single operator for each, and intra-examiner reliability was evaluated (kappa scores of 0.85 and 0.80 respectively).

In the present study failure loads for dentin bonded ceramic crowns luted with Bioactive, resin and GIC cements ranged in 350-540 N, 500-700 N and 200-350N respectively. Clinically, the average masticatory force at swallowing is 40N, however the maximum mean occlusal forces range between 200 to 540N, as reported in earlier studies.^17^-^19^ In association with these standards, dentin bonded crowns luted with GIC cannot be used clinically. By contrast, dentin bonded ceramic crowns can be used as a reliable restorative treatment option, due to their ability of dentin bonding and fracture resistance. Similar findings have been shown in previous studies.^20^,^21^ A resin cement containing multiple methacrylate monomer/polymer (Bis-GMA and TEGDMA), interacts well with the silanized ceramic surface and primed dentin. They result in forming a monobloc of the ceramic-cement-tooth reconstruction therefore enforcing the ceramic and enhancing its fracture loads.^13^ Glass ionomers on the other hand fail to show clinically reliable failure loads, this is attributed to the comparative inferior mechanical properties and failure to bond to silanized ceramic surface.^22^,^23^

Interestingly, failure loads for specimen cemented with Bioactive cement (350-540N) were either below or in the range of acceptable maximum masticatory loads (200-540N). In a study by Grin et al., Bioactive materials have shown compressive and tensile strengths comparable to resin based materials.^11^ Therefore a possible explanation for lower failure loads could be the hydrophilic nature of bioactive cement.^24^ This may result in water absorption and release, resulting in interfacial breakdown within the ceramic tooth complex. Bioactive cement (ACTIVA) should be considered as a hybrid material with biological and mechanical properties similar to GIC and resin respectively. In addition, resin cement interacts closely with ceramic silane molecules, however the same is true for Bioactive cement is not known. This hypothesis is supported by the fact that the specimens cemented with GIC showed complete fracture and delamination of ceramic from the dentin surface, however the same was not true for bioactive-luted specimens. It was also observed that the failure loads significantly reduced for bioactive cement in comparison to resin cement due to thermocycling in the present study. Thermocycling repeatedly exposes the specimens to temperature changes in a moist environment. Hydrophilic monomers, like the same in Bioactive cements are known to uptake and release ions and water molecules resulting in hygroscopic expansion and contraction.^25^ These events may disrupt the ceramic cement interface causing failure load compromise in bioactive-cemented samples as shown in the present study.

From a clinical perspective, resin cement is still the gold standard for luting dentin bonded all ceramic crowns for reliable long-term clinical prognosis. Bioactive cements (ACTIVA) can be employed for cementation of dentin bonded crowns and veneers in anterior teeth (low occlusal loads). However, these outcomes should be considered in light of the possible limitations.

### Limitatis of the study

The study included bonding of dentin bonded crowns to natural tooth dentin. Dentin structure varies from tooth to tooth and location within a tooth along with the structure of hydroxyapatite. In addition, the load application on luted crowns was axial and rapid, in contrast to the clinical occlusal load, which are non-axial and vary in duration. The outcomes of the study should only be attributed to the materials and techniques used and cannot be generalized. Therefore, further prospective randomized controlled trials assessing the influence of Bioactive cements on the fracture resistance and clinical function of dentin bonded restorations are recommended.

## CONCLUSION

Within the limitation of this study, dentin boned ceramic crowns cemented with resin cement showed higher failure loads than Bioactive and GIC luted crowns and are suitable for restoration of posterior teeth. Crowns luted with Bioactive cement showed acceptable failure loads for use as restoration on anterior teeth.

### Authors’ Contribution:

**FV:** Data collection, study design, manuscript writing, final manuscript approval and is responsible for the accuracy and integrity of the work.

**TA:** Data collection, study design, manuscript drafting, data analysis, manuscript approval.

**MA & AA:** Specimen design and preparation, Data collection, manuscript approval and data interpretation.

**MAR & MAD:** Data collection, writing, revise, editing and final manuscript approval

**YF & KA:** Study design, statistical analysis, data interpretation, manuscript writing, table and figure designing.
